# Heterologous complementation of a *pyrF* deletion in *Caldicellulosiruptor hydrothermalis* generates a new host for the analysis of biomass deconstruction

**DOI:** 10.1186/s13068-014-0132-8

**Published:** 2014-09-16

**Authors:** Joseph Groom, Daehwan Chung, Jenna Young, Janet Westpheling

**Affiliations:** Department of Genetics, University of Georgia, Athens, GA 30602 USA; The BioEnergy Science Center, Oak Ridge National Laboratory, Oak Ridge, TN USA

## Abstract

**Background:**

Members of the thermophilic, anaerobic Gram-positive bacterial genus *Caldicellulosiruptor* grow optimally at 65 to 78°C and degrade lignocellulosic biomass without conventional pretreatment. Decomposition of complex cell wall polysaccharides is a major bottleneck in the conversion of plant biomass to biofuels and chemicals, and conventional biomass pretreatment includes exposure to high temperatures, acids, or bases as well as enzymatic digestion. Members of this genus contain a variety of glycosyl hydrolases, pectinases, and xylanases, but the contribution of these individual enzymes to biomass deconstruction is largely unknown. *C. hydrothermalis* is of special interest because it is the least cellulolytic of all the *Caldicellulosiruptor* species so far characterized, making it an ideal naïve system to study key cellulolytic enzymes from these bacteria.

**Results:**

To develop methods for genetic manipulation of *C. hydrothermalis*, we selected a spontaneous deletion of *pyrF,* a gene in the pyrimidine biosynthetic pathway, resulting in a strain that was a uracil auxotroph resistant to 5-fluoroorotic acid (5-FOA). This strain allowed the selection of prototrophic transformants with either replicating or non-replicating plasmids containing the wild-type *pyrF* gene. Counter-selection of the *pyrF* wild-type allele on non-replicating vectors allowed the construction of chromosomal deletions. To eliminate integration of the non-replicating plasmid at the *pyrF* locus in the *C. hydrothermalis* chromosome, we used the non-homologous *Clostridium thermocellum* wild-type *pyrF* allele to complement the *C. hydrothermalis pyrF* deletion. The autonomously replicating shuttle vector was maintained at 25 to 115 copies per chromosome. Deletion of the ChyI restriction enzyme in *C. hydrothermalis* increased the transformation efficiency by an order of magnitude and demonstrated the ability to construct deletions and insertions in the genome of this new host.

**Conclusions:**

The use of *C. hydrothermalis* as a host for homologous and heterologous expression of enzymes important for biomass deconstruction will enable the identification of enzymes that contribute to the special ability of these bacteria to degrade complex lignocellulosic substrates as well as facilitate the construction of strains to improve and extend their substrate utilization capabilities.

**Electronic supplementary material:**

The online version of this article (doi:10.1186/s13068-014-0132-8) contains supplementary material, which is available to authorized users.

## Introduction

Plant biomass recalcitrance is one of the most important barriers to its use as a substrate for the production of fuels and chemicals using microorganisms as catalysts. The plant cell wall consists of a complex web of polysaccharides and phenolics that function in plant structure and development [1]. Perennial plants like switchgrass could be incorporated into so-called agro-ecosystems, which would increase carbon storage and biofuel production, decrease carbon dioxide emissions, and improve water quality through wetland denitrification [2]. While the natural recalcitrance of plant biomass is a major barrier [3], several methods including direct microbial breakdown of cell wall structures can be used to liberate energy-rich sugars for conversion to useful biofuels and bioproducts.

Chemical and thermal pretreatments are often used to break down the raw substrate, but they are expensive and destructive to the sugars in the biomass [4], and they produce hydrolysates inhibitory to both cellulose degradation and microbial growth [5]. In contrast, thermophilic anaerobes in the genus *Caldicellulosiruptor* can deconstruct high loadings of plant biomass into simple sugars without conventional pretreatment [6-8] and have recently been engineered to produce ethanol directly from switchgrass [9]. *Caldicellulosiruptor* species can simultaneously utilize the wide range of hexoses, pentoses, oligosaccharides, and polysaccharides released from the plant cell wall, and there is no evidence of carbon catabolite repression [10,11]. These qualities make them well suited for consolidated bioprocessing (CBP), in which one microorganism is used for both biomass deconstruction and end-product formation.

Members of the *Caldicellulosiruptor* genus are anaerobic Gram-positive bacteria, and they are the most thermophilic cellulose-degrading organisms known [12]. They secrete free carbohydrate-active enzymes (CAZys) [13] with carbohydrate-binding modules that are well suited for binding and degrading cell wall polysaccharides [14]. *C. bescii* is one of the strongest crystalline cellulose degraders in the genus, whereas the closely related *C. hydrothermalis* is one of the weakest [7]. Interestingly, *C. hydrothermalis* lacks the multidomain CAZys found in more cellulolytic members of the genus [15] as well as a cluster of pectinases that affect *C. bescii* growth on biomass (D. Chung, submitted). In addition to lacking multidomain enzymes, *C. hydrothermalis* completely lacks the glycosyl hydrolase (GH) domains GH9 and GH48 [12], the domains that comprise CelA, the most highly secreted cellulase in *C. bescii* [16]. *C. hydrothermalis* thus provides a “blank slate” with which to study thermophilic enzymes important for biomass degradation *in vivo*, and is a promising system for heterologous expression of plant biomass deconstruction enzymes.

To establish methods for genetic manipulation of *C. hydrothermalis*, we took an approach similar to the one previously used for *C. bescii* that relied on the selection of a *pyrF* deletion mutant, which allows for nutritional selection of transformants [17]. Interestingly, *C. hydrothermalis* contains fewer mobile genetic elements than other members of the genus [18], so this species may have other advantages for genetic manipulation, including fewer issues with genome stability that could result from genetic selections and counter-selections. We transformed the *pyrF* deletion mutant with the pJGW07 shuttle vector that is based on a native plasmid, pBAS2, from *C. bescii* [19]. Both the *C. bescii* and the heterologous *Clostridium thermocellum* wild-type *pyrF* allele were shown to complement this deletion, restoring the mutant to uracil prototrophy. Deletion of the ChyI restriction enzyme in *C. hydrothermalis*, a homolog of a HaeIII-like restriction enzyme known to be a barrier to transformation in *C. bescii* [20,21], increased the transformation efficiency by about an order of magnitude. The new strain *C. hydrothermalis* JWCH008 should facilitate the assessment of plant biomass deconstruction by the *Caldicellulosiruptor* genus and the molecular engineering of deconstruction enzymes.

## Results and discussion

### Selection for resistance to 5-FOA resulted in a spontaneous deletion of the *pyrF* gene in *C. hydrothermalis*

**Figure 1:**
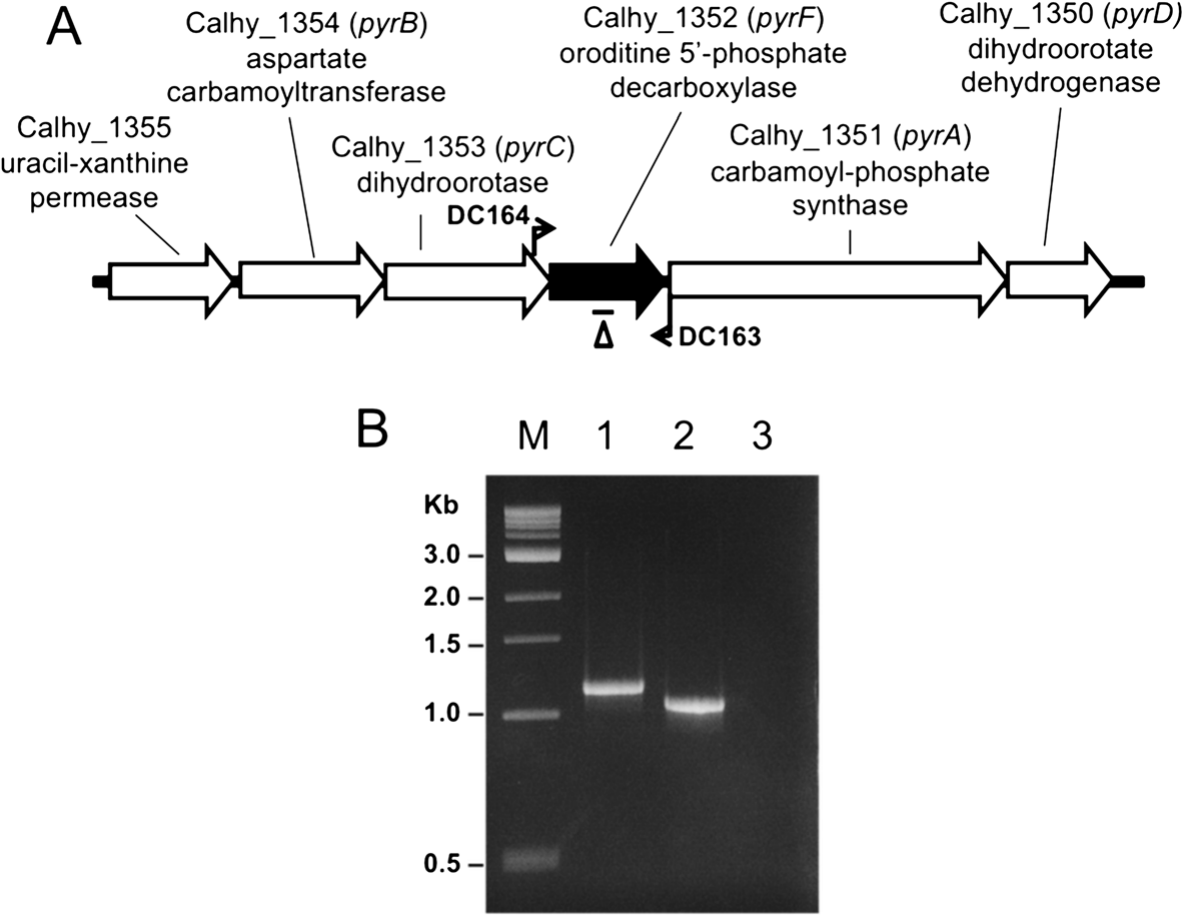
**Isolation of a spontaneous** ***pyrF*** **mutant in** ***C. hydrothermalis*** **. (A)** Chromosomal map of the uridine monophosphate (UMP) biosynthetic gene cluster in *C. hydrothermalis* JWCH006. The 99-bp spontaneous deletion in *∆pyrF* is indicated by the line below the diagram. 462 bp lie upstream and 357 bp lie downstream of the deletion. Bent arrows depict primers used to verify the structure of the *pyrF* gene in the JWCH006 strain. **(B)** Gel depicting PCR products of the *pyrF* region in the wild-type strain (1.13 kb) compared to the JWCH006 strain (1.02 kb) amplified by the indicated primers (DC163 and DC164). M: 1 kb DNA ladder (NEB); 1: wild-type genomic DNA; 2: JWCH006 genomic DNA; 3: negative control.

The *pyrF* gene encodes orotidine monophosphate decarboxylase, an enzyme in the pyrimidine biosynthesis pathway. Deletion of this gene results in uracil auxotrophy and resistance to 5-fluoroorotic acid (5-FOA), allowing prototrophic selection of transformants and counter-selection of the wild-type allele [22]. The optimal growth temperature for *C. hydrothermalis* is 65°C [23], and we had previously observed an increase in the spontaneous mutation rate in cells grown above and below 65°C. To obtain spontaneous deletions of *pyrF*, cells were grown at non-optimal temperatures in the presence of uracil. The presence of uracil in the growth medium allowed for the maintenance of cells with spontaneous mutations in the *pyrF* gene. After growth at various temperatures, cells were plated onto a medium with 5-FOA selecting resistance and loss of *pyrF* function. One 5-FOA resistant mutant, JWCH006 (Table 1), that had been grown at 60°C contained a 99-bp deletion in *pyrF* (Figure 1A) and was confirmed to be a uracil auxotroph resistant to 5-FOA.

**Table 1:** **Strains and plasmids used in this study**

**Strain or plasmid**	**Genotype/phenotype**
JWCH001	*C. hydrothermalis* DSM 18901 wild -type (*ura* ^*+*^ */*5-FOA^S^)
JWCH006	*C. hydrothermalis ΔpyrF* (*ura* ^*−*^ */*5-FOA^R^)
JWCH008	*C. hydrothermalis ΔpyrF ΔchyI* (*ura* ^*−*^ */*5-FOA^R^)
JWCH009	*C. hydrothermalis ΔpyrF* harboring pJGW07 (*ura* ^*+*^ */*5-FOA^S^)
JW401	DH5α containing pJGW03 (Apramycin^R^)
JW402	DH5α containing pJGW07 (Apramycin^R^)
pDCW89	*E. coli/Caldicellulosiruptor* shuttle vector (*C. bescii pyrF*)
pDCW88	*Caldicellulosiruptor* non-replicating vector (*C. bescii pyrF*)
pDCW151	*Caldicellulosiruptor chyI* deletion vector (*C. bescii pyrF)*
pJGW03	*Caldicellulosiruptor chyI* deletion vector (*C. thermocellum pyrF)*
pJGW07	*E. coli/Caldicellulosiruptor* shuttle vector (*C. thermocellum pyrF*)

### Uracil auxotrophy in *C. hydrothermalis* is complemented by heterologous expression of the *Clostridium thermocellum pyrF* gene

Electrocompetent *C. hydrothermalis* JWCH006 (*ΔpyrF*) cells were prepared based on the method for *C. bescii* [17] and transformed with a previously described shuttle vector, pDCW89 [24], containing the wild-type *C. bescii pyrF* allele. Transformation of *C. bescii* with plasmid DNA isolated from *E. coli* requires *in vitro* methylation by a methyltransferase, M.CbeI [17]. As *C. hydrothermalis* has a similar restriction profile to that of *C. bescii* [20], we anticipated that *in vitro* methylation by M.CbeI would protect plasmid DNA isolated from *E. coli* and allow transformation of *C. hydrothermalis*. pDCW89 DNA methylated with M.CbeI *in vitro* successfully transformed the *C. hydrothermalis ΔpyrF* mutant to prototrophy at an average frequency of 37 colony forming units (CFUs) per microgram of DNA (Table 2).

**Table 2:** **Transformation efficiency (CFU/μg DNA)**

**Strain**	**pJGW07**	**pJGW07M**	**pDCW89**	**pDCW89M**
**JWCH006 Δ** ***pyrF***	33 ± 24	22 ± 10	18 ± 20	37 ± 43
**JWCH008 Δ** ***pyrF*** **Δ** ***chyI***	395 ± 301	241 ± 174	58 ± 48	100 ± 90

The suffix M denotes plasmid methylated with *C. bescii* M.CbeI methyltransferase.

Results represent the average ± standard deviation of three biologically independent transformation experiments.

**Figure 2:**
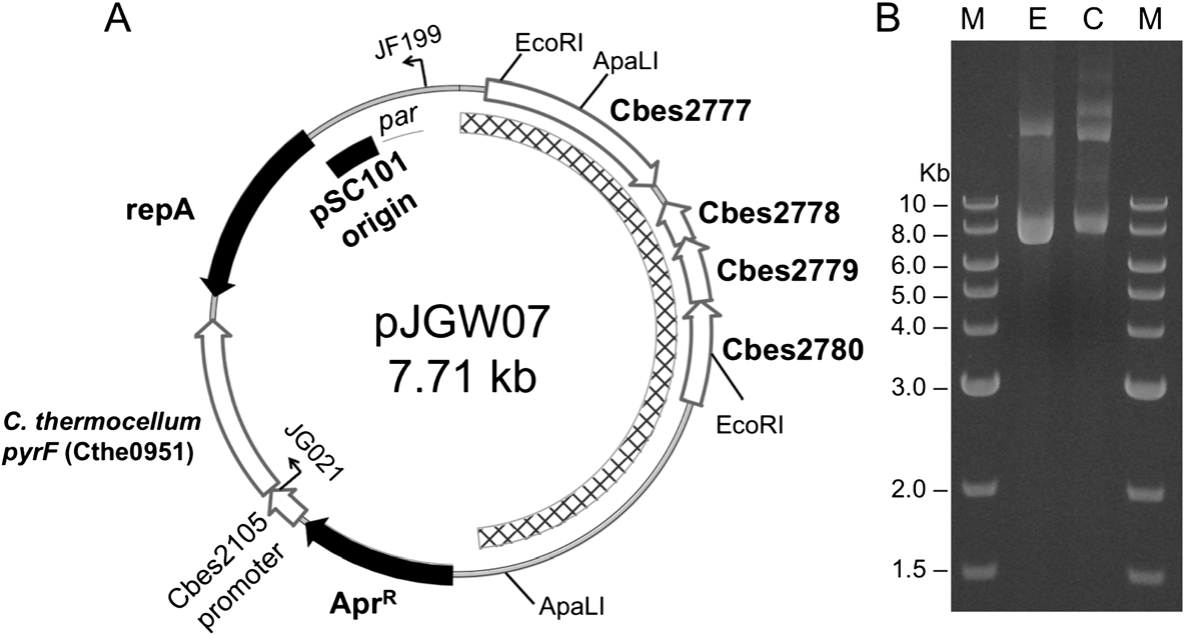
**Plasmid pJGW07 isolated directly from** ***C. hydrothermalis*** **Δ** ***pyrF*** **. (A)** pJGW07 constructed by replacing the *C. bescii pyrF* gene with the *C. thermocellum* ATCC 4705 homolog Cthe0951. The hatched region was derived from *C. bescii* native plasmid pBAS2. Apr^R^, apramycin resistance casette; *repA*, replication initiator for pSC101 replication origin; *par*, partitioning locus. **(B)** Agarose gel depicting pJGW07 plasmid DNA extracted from different sources. M, molecular weight standards; E, pJGW07 purified from *E. coli*; C, pJGW07 purified from *C. hydrothermalis.*

Since the deletion of *pyrF* in JWCH006 was only 99 bp (Figure 1A), and there is 95% DNA sequence homology between the *pyrF* genes in *C. bescii* and *C. hydrothermalis*, there was a large region of residual homology between the *pyrF* locus in the *C. hydrothermalis* chromosome and the *C. bescii pyrF* gene on the plasmid. Although pDCW89 DNA methylated *in vitro* with M.CbeI transformed *C. hydrothermalis* JWCH006 to prototrophy, we found that the wild-type plasmid-encoded *pyrF* allele repaired the mutant *pyrF* locus in almost every case (Additional file 1: Figure S1), and the plasmid was not maintained autonomously over time. Since marker replacement in *C. hydrothermalis* relies on the integration of non-replicating plasmids at sites other than *pyrF*, the *C. bescii pyrF* cassette in pDCW89 was replaced by the *C. thermocellum* homolog (Cthe0951) to create pJGW07 (Figure 2A). While the two PyrF protein sequences are 45% identical, the *C. thermocellum pyrF* gene has very low DNA sequence homology with the *Caldicellulosiruptor pyrF* genes. Transformation of JWCH006 with methylated pJGW07 was successful (Figure 2B, Additional file 1: figure S2A), and uracil prototrophic transformants were obtained at a frequency of 22 ± 10 CFU per microgram of DNA (Table 2).

Plasmid pJGW07 was purified directly from *C. hydrothermalis* based on a previously published method for other Gram-positive organisms [25]. Undigested plasmid isolated from *C. hydrothermalis* migrated in an agarose gel slightly differently from plasmid DNA isolated from *E. coli*, and we suggest that the difference is not in size but in methylation within the native host compared to *E. coli* (Figure 2B). Endonuclease restriction analysis using a panel of enzymes indicated that plasmid isolated from *C. hydrothermalis* is protected at GGCC sites by a HaeIII-like modification system as expected but not at GATC sites (Additional file 1: Figure S3). This suggests that *C. hydrothermalis* lacks the DNA adenine methylase present in *E. coli*. EcoRI or HhaI recognition sites are not protected in either organism.

To confirm that the plasmid was replicating autonomously, DNA isolated from the *C. hydrothermalis* transformant JWCH009 (Table 1) was back-transformed into *E. coli* DH5*α*. Plasmid DNA recovered from 12 apramycin-resistant *E. coli* transformants was identical in its restriction patterns to pJGW07 transformed into *C. hydrothermalis,* suggesting that the plasmid was structurally stable during transformation, replication, and back-transformation to *E. coli* (Additional file 1: Figure S2). There was no evidence of plasmid integration (Additional file 1: Figure S4) resulting from recombination between the *C. thermocellum pyrF* gene on the plasmid and the *C. hydrothermalis pyrF* gene in the chromosome.

These data show that, although *C. thermocellum* has an optimal growth temperature of 60°C, the *C. thermocellum* orotidine-5′-phosphate decarboxylase functions at temperatures up to at least 65°C and that the *C. thermocellum* gene is expressed at sufficient levels to complement the *C. hydrothermalis pyrF* deletion.

### A shuttle vector derived from a native *C. bescii* plasmid is maintained at a high copy number in *C. hydrothermalis*

**Figure 3:**
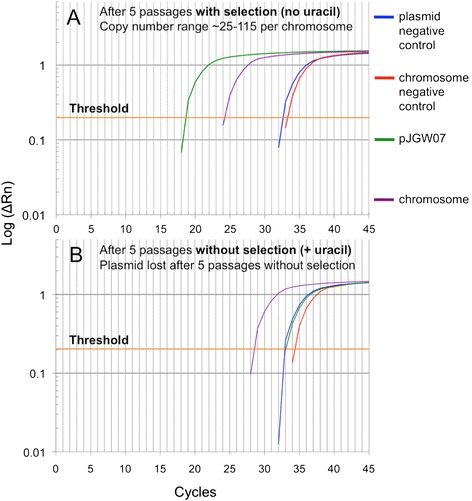
**Determination of plasmid copy number.** Quantitative PCR (qPCR) was used to detect the copy number of plasmid pJGW07 in relation to the chromosome. Shown are the results after the fifth passage through **A)** selective and **B)** non-selective media. The *x*-axis is the number of iterations of the polymerase chain reaction, and the *y*-axis displays the logarithm of ΔRn, which is the fluorescence of the SYBR green dye with the baseline fluorescence subtracted. The number of cycles required to cross a given threshold (cycles to threshold or Ct) is reflective of the plasmid copy number (PCN). The threshold is indicated by a horizontal line. PCN was calculated using the formula PCN = 2^| Ct_chromosome_— Ct_plasmid_ |. The copy number ranged from about 25 to 115 copies per chromosome in the cultures with selective media. The copy number was determined based on two independent biological samples with three technical replicates (Additional file 1: Table S1).

We recently reported the construction of a shuttle vector for *C. bescii* [24] based on the smaller of two native plasmids in that species [19]. The native plasmid pBAS2 is maintained in *C. bescii* at a copy number of approximately 75 [24]. Because the shuttle vector derived from this plasmid competed with the native plasmid it was derived from, the shuttle vector was maintained in low copy and was readily lost without selection. While an unstable plasmid is useful for some applications, stability and high copy number also have advantages. No native plasmid DNA was detected in *C. hydrothermalis* using conventional plasmid isolation methods, nor was one identified during the sequencing of total DNA isolated from this strain [26]. We anticipated that the *C. bescii* shuttle vector would likely replicate in *C. hydrothermalis* and might be stably maintained at a high copy number. Quantitative polymerase chain reaction (qPCR) was performed as described in the Methods section to determine the copy number of the pJGW07 plasmid in *C. hydrothermalis*, and the results indicated that it was maintained between 25 and 115 copies per chromosome over five serial transfers, but was quickly lost without selection (Figure 3, Additional file 1: Table S1).

### Deletion of the *C. hydrothermalis* ChyI restriction enzyme results in increased transformation efficiency

**Figure 4:**
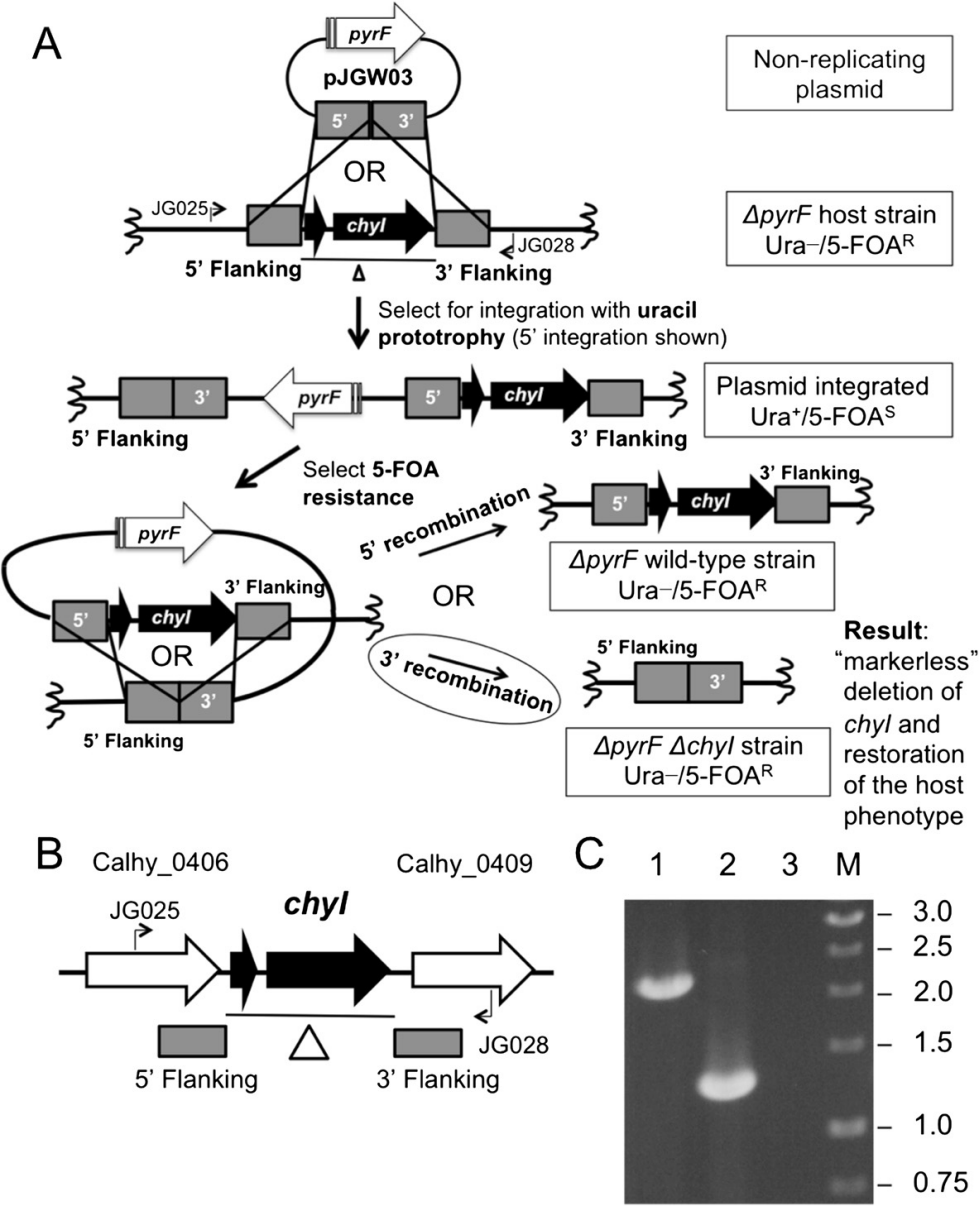
**Deletion of the gene encoding putative restriction enzyme ChyI. A)** Scheme for targeted gene deletion. The pJGW03 *chyI* knockout vector is transformed into JWCH006 (*ΔpyrF*), and uracil prototrophy selects for integration at one of the 500-bp flanking regions, denoted by the gray boxes. 5′ integration is shown. The uracil prototroph is then plated on 5-FOA to select for loop-out of the *pyrF* cassette via homologous recombination between flanking region sequences. The two possible results are a return to the wild-type sequence or a clean *chyI* deletion. **(B)** Chromosomal map of the locus containing the ORF for *chyI*. The deleted region is indicated by the line below the diagram. Bent arrows depict primers used to verify deletion of *chyI* in the JWCH008 strain. **(C)** Gel depicting PCR products of the *chyI* region in the JWCH006 ∆*pyrF* strain (2.3 kb) compared to the JWCH008 ∆*pyrF* ∆*chyI* strain (1.25 kb) amplified by the indicated primers (JG025 and JG028). 1: *C. hydrothermalis* JWCH006 genomic DNA; 2: *C. hydrothermalis* JWCH008 genomic DNA; 3: negative control; M: 1 kb DNA ladder (NEB).

In developing methods for DNA transformation of *C. bescii*, we observed that restriction by a HaeIII isoschizomer, CbeI [21], was an absolute barrier to transformation of DNA from *E. coli*. We identified, cloned, expressed, and purified its cognate methyltransferase, M.CbeI, from *C. bescii* and showed that DNA methylated *in vitro* readily transformed *C. bescii* [17]. Deletion of *cbeI* in *C. bescii* relieved the requirement for *in vitro* methylation of plasmid DNA from *E. coli* by M.CbeI [20] and allowed efficient DNA transformation. The *C. hydrothermalis chyI* gene is an ortholog of *cbeI* with 96% DNA sequence identity and 100% sequence coverage*.* A deletion of *chyI* was constructed on a plasmid, pJGW03, which was transformed into *C. hydrothermalis* JWCH006 (Figure 4A). A deletion mutant was readily obtained in a screen of 50 colonies, and only two rounds of purification on low osmolarity defined (LOD) plating media containing uracil [27] were required to resolve the merodiploid, resulting in *C. hydrothermalis* JWCH008 *(ΔpyrF ΔchyI*) (Figure 4C). We note that the region of the *C. hydrothermalis* genome that contains ChyI is not identical to the region of the *C. bescii* chromosome that contains CbeI. The *C. hydrothermalis* region contains an additional open reading frame (ORF) that apparently encodes a truncated form of the N-terminal portion of the ChyI protein. Sequence analysis revealed a premature stop codon likely resulting from a point mutation in the ChyI ORF. This altered gene structure had no obvious effect on enzyme function *in vivo*. The deletion we designed included both ORFs.

To assess whether the loss of the ChyI restriction enzyme resulted in an increase in transformation efficiency in *C. hydrothermalis*, we compared the transformation efficiencies of the two strains generated in this study, JWCH006 (*ΔpyrF*) and JWCH008 (*ΔpyrF ΔchyI*). As shown in Table 2, electrotransformation of the JWCH006 parent strain with either the pJGW07 shuttle vector containing the *C. thermocellum pyrF* gene, or the pDCW89 vector containing the *C. bescii pyrF* gene, was low but detectable, and methylation of plasmid DNA did not make a significant difference in transformability. The standard deviation in experiments with low numbers of transformants is substantial but not unexpected [28]. This extremely low transformation efficiency may be an underestimate of the actual efficiency, as the plating efficiency of *C. hydrothermalis* on a selective solid medium is less than 10^−4^ (plating 10^6^ cells as determined by cell count resulted in fewer than 100 colonies). Transformation of JWCH008 containing the *chyI* deletion was an order of magnitude higher than that of JWCH006, and again, there is no significant difference between the transformation efficiency of methylated and unmethylated DNA, suggesting differences in the restriction/modification systems of *C. hydrothermalis* and *C. bescii*. The fact that methylation with M.CbeI made no difference in transformation efficiency was somewhat surprising, especially considering the fact that deletion of ChyI increased the transformation frequency of unmethylated DNA. We interpret this to indicate that there are differences between *C. bescii* and *C. hydrothermalis* in their restriction/modification systems and perhaps additional enzymes in one or the other that account for the differences in transformation frequencies. It is also possible that the truncated form of the CbeI/ChyI orthologous proteins makes a difference in their activities (Figure 4B). We previously reported that *C. hydrothermalis* chromosomal DNA is resistant to digestion by BamHI and BspEI [20]. Both these enzymes have six base recognition sequences that are relatively rare compared to four base recognition sequences. The plasmids used in this study do not contain BamHI sites. While there are two BspEI restriction sites (TCCGGA), *E. coli* DH5α, the strain we used to make plasmid DNA, contains an adenine methyltransferase known to protect this site and may prevent cleavage by *C. hydrothermalis* during DNA transformation.

We emphasize that the observed transformation of the JWCH006 parent strain is not an indication that *C. hydrothermalis* is naturally competent for DNA uptake. Preparation of electrocompetent cells and electroporation was necessary to detect transformation. We have invested some effort to induce natural competence in members of this genus, but those efforts have not been successful. There are homologs to the natural competence genes *comEA, comEC, comGC,* and *dprA* [29], but in contrast to other thermophilic anaerobes [30], there is no evidence to date of natural competence in *Caldicellulosiruptor* species.

## Conclusions

Methods for genetic manipulation of *C. hydrothermalis,* based on those used for *C. bescii,* were successful and efficient. Restriction of DNA was not an absolute barrier to transformation, but deletion of the ChyI restriction enzyme in *C. hydrothermalis* increased the transformation efficiency by an order of magnitude. Heterologous expression of the *Clostridium thermocellum pyrF* gene was sufficient to complement the *C. hydrothermalis ΔpyrF* mutant, allowing both autonomous plasmid replication at relatively high copy (about 25 to 115 copies/chromosome) and marker replacement of the *chyI* gene in the *C. hydrothermalis* chromosome. The use of this new strain, *C. hydrothermalis* JWCH008, should allow for the expression of heterologous and homologous enzymes for both the identification and analysis of enzymes involved in biomass deconstruction of unpretreated plant biomass by the *Caldicellulosiruptor* genus. It will also enable the engineering of glycosyl hydrolases such as CelA and other important plant biomass deconstruction enzymes in a strain devoid of similar enzymes or activities.

## Methods

### Selection of a *pyrF* deletion

Wild-type *C. hydrothermalis* DSM 18901 was grown from a 0.5% inoculum in 50 mL of a low osmolarity defined growth medium (LOD) supplemented with 40 μM uracil. Cultures were grown at 55°C, 60°C, 68°C, and 75°C. Cells in the late exponential phase were cooled to room temperature, harvested by centrifugation at 6,100 × g, and resuspended in 1X *C. bescii* partial base salts [6]. 100 μL of resuspended cells were mixed with a 1.5% agar overlay and plated onto LOD media with 40 μM uracil and 8 mM 5-FOA. The plates were degassed in anaerobic chambers and incubated for 4 days at 68°C. The colonies were picked directly into 20 mL LOD media with 40 μM uracil, which was immediately degassed and incubated at 68°C. When the media was turbid, chromosomal DNA was extracted with a Zymo Research gDNA Extraction kit (Irvine, CA). The *pyrF* gene region was amplified from the wild type and deletion mutant with primers DC163 and DC164 (Figure 1A), and the PCR products were analyzed for the presence of deletions in a 1.5% w/v agarose gel by electrophoresis (Figure 1B). All plasmids were verified by DNA sequencing (GENEWIZ).

### Construction of a shuttle vector containing the *pyrF* gene from *Clostridium thermocellum* ATCC 27405

High-Fidelity Q5 Polymerase (New England Biolabs (NEB), Ipswich, MA), restriction enzymes (NEB), and Fast-Link™ DNA Ligase (Epicentre Technologies, Madison, WI) were used according to manufacturer instructions. pJGW07 (Figure 2A) was constructed by replacing the *C. bescii pyrF* gene (Cbes1377) on the replicating shuttle vector pDCW89 [24] with the *Clostridium thermocellum* homolog for *pyrF* (Cthe0951). Cthe0951 was amplified by PCR using primers JG021 and JG022, engineered to contain XbaI and NcoI restriction sites, respectively. This 945-bp PCR product was ligated directionally to a 6.75-kb pDCW89 PCR product amplified using primers JG023 and JG024, which also contain XbaI and NcoI sites. Correct clones were purified from *E. coli* with a Miniprep kit (Qiagen), and confirmed by restriction fragment analysis and DNA sequencing (GENEWIZ).

### Transformation of *C. hydrothermalis* JWCH006

To prepare cells for transformation, 15 mL of a freshly grown JWCH006 (*ΔpyrF*) culture were inoculated into four 500-mL bottles of fresh LOD supplemented with 40 μM uracil and 19 amino acids, and incubated at 65°C to the early exponential phase (OD_680_ approximately 0.04 to 0.05). The cultures were cooled to room temperature for 1 h, harvested by centrifugation (5,000 × g, 10 min) at 4°C and washed three times with 50 mL pre-chilled 10% sucrose. After the final wash, the cell pellets were resuspended in about 250 μL pre-chilled 10% sucrose. 60-μL aliquots of competent JWCH006 were added to plasmid DNA (0.5 μg), either methylated with M.CbeI methyltransferase, as previously described [17], or unmethylated, gently mixed, and incubated in 10% sucrose for 15 min at room temperature. Electrotransformation of the cell/DNA mixture was performed via a single exponentially decaying electric pulse (1.8 kV, 350 *Ω*, and 25 microF) in a pre-chilled 1-mm cuvette using a Bio-Rad Gene Pulser (Bio-Rad, Hercules, CA). After pulsing, the cells were incubated in 10 mL low osmolarity complex growth medium (LOC) [27] at 65°C. After 4 h, the cultures growing in LOC medium were cooled to room temperature, harvested by centrifugation (6,100 × g, 10 min), and washed three times at room temperature with 1X *C. bescii* partial base salts [6] to remove the rich media. The cells were finally resuspended in 800 μL 1X base salts. For each plate, 100 μL of resuspended cells were mixed with 2 mL of a 1% agar overlay and plated onto LOD media lacking uracil to select for transformation. The plates were degassed in anaerobic chambers and incubated for 4 days at 65°C. The colonies were picked directly into 20 mL LOD media without uracil, which was immediately degassed and incubated at 65°C. Uracil-prototrophic transformants were confirmed by PCR amplification of *C. hydrothermalis* DNA using primers JG021 and JF199, which are specific for pJGW07 (Figure 2C, Additional file 1: figure S2A).

### Analysis of plasmid structure and stability in *C. hydrothermalis*

A single transformant colony was picked directly into 20 mL LOD media lacking uracil, which was immediately degassed and incubated at 65°C. This strain maintaining the pJGW07 shuttle vector was named JWCH009. JWCH009 was grown to the late exponential phase (OD_680_ approximately 0.15) in 50 mL LOD. Direct extraction of plasmid DNA from JWCH009 was performed as previously described [21,25]. The plasmid DNA was digested with enzymes HaeIII, EcoRI, HhaI, and MboI (NEB).

To determine the structural stability of the plasmid, DNA was extracted from *C. hydrothermalis* with a gDNA Extraction kit (Zymo Research), and 2 μL of DNA was electrotransformed into *E. coli* DH5α via single electric pulse (2.5 kV, 200 *Ω*, and 25 microF) in a pre-chilled 2-mm cuvette using a Bio-Rad Gene Pulser. The cells were placed into 1 mL SOC media for 1 h with shaking at 37°C, and then plated onto LB agar supplemented with 50 μg/mL apramycin. The colonies were picked into 10 mL LB with 50 μg/mL apramycin, and the plasmid DNA was extracted using a Miniprep kit (Qiagen) and screened with restriction enzymes EcoRI and ApaLI (NEB) (Additional file 1: Figure S2B).

### Plasmid copy number determination by quantitative PCR

To determine the pJGW07 copy number and maintenance over time, JWCH009 was grown to OD_680_ approximately 0.15 and serially subcultured for 5 days. The total DNA was isolated from the cultures and treated with RNase A. qPCR experiments were carried out with a LightCycler 480 Real-Time PCR instrument (Roche, Indianapolis, IN) with LightCycler 480 SYBR Green I Master mix (Roche). Two independent sets of primers specific to either the pJGW07 plasmid (Q1/Q2 inside Cbes2777, Q3/Q4 inside Cbes2778) or the *C. hydrothermalis* chromosome (Q11/Q12 inside Calhy0897, Q13/Q14 inside Calhy1377) were used to compute the relative copy number of the plasmid to the chromosome. Three replicate reactions for each primer set were performed, and the average of the two primer sets on each sample was used to calculate the plasmid copy number in each serial subculture (Additional file 1: Table S1) according to the method of Lee *et al.* [31]. The amplification efficiency over a 10^4^-fold range of DNA concentrations was 93.5%, within the ratio of 90 to 110% considered acceptable (Life Technologies).

### Construction of a deletion of the *chyI* gene in *C. hydrothermalis*

A vector backbone was amplified from suicide vector pDCW88 [20] with primers DC081 and DC262. Flanking regions for the *chyI* gene (Calhy0408) were amplified using primers DC484 and DC485 (5′ flanking region) and DC486 and DC487 (3′ flanking region). The 5′ and 3′ flanking regions were combined into one fragment by overlap extension PCR and ligated into the pDCW88 vector backbone using restriction enzymes KpnI and ApaLI. The resulting plasmid pDCW151 was used to construct pJGW03 in which the *C. bescii pyrF* gene cassette (Cbes1377) was replaced with the *C. thermocellum pyrF* gene (Cthe0951) as described above. Competent *C. hydrothermalis* JWCH006 cells were prepared as described above. 1.0 μg of non-replicating plasmid pJGW03 was added to 50-μL aliquots of competent JWCH006, gently mixed, and incubated for 15 min at room temperature. Electrotransformation of the cell/DNA mixture was performed via a single electric pulse (1.8 kV, 350 *Ω*, and 25 microF) in a pre-chilled 1-mm cuvette using a Bio-Rad Gene Pulser. After pulsing, the cells were incubated in 10 mL low osmolarity complex growth medium (LOC) [27] at 68°C with shaking at 150 rpm. From this culture, 0.5% inocula were transferred into LOD medium lacking uracil at 65°C every hour for 4 h to select for integration into the *C. hydrothermalis* genome. When the cultures in the selective media were turbid, a 0.5% inoculum was transferred to 50 mL LOD supplemented with 40 μM uracil to allow a loopout of the wild-type *pyrF* allele to occur. This culture was then plated onto LOD plates supplemented with 40 μM uracil and 6 mM 5-FOA to select against the *pyrF* wild-type allele, and grown for 4 days at 68°C. Colonies were picked directly into 20 mL LOD medium with uracil, which was immediately degassed and incubated at 68°C. The resulting cultures were screened for a deletion using primers JG025 and JG026, and one deletion culture was purified twice on solid LOD media supplemented with 40 μM uracil.

## Additional file

Additional file 1: Figure S1. Repair of the *pyrF* gene in pDCW89 transformants. **Figure S2.** Evidence for transformation and stable replication of the *Caldicellulosiruptor/E. coli* shuttle vector pJGW07 in *C. hydrothermalis.* **Figure S3.** Restriction digest analysis of pJGW07. **Figure S4.** Maintenance of the mutated *pyrF* gene in pJGW07 transformants. **Table S1.** Quantitative PCR data. **Table S2.** Primers used in this study.

## References

[1] Albersheim P, Darvill A, Roberts K, Sederoff R, Staehelin A (2011). Plant Cell Walls.

[2] Jordan N, Boody G, Broussard W, Glover JD, Keeney D, McCown BH, McIsaac G, Muller M, Murray H, Neal J, Pansing C, Turner RE, Warner K, Wyse D (2007). Environment. Sustainable development of the agricultural bio-economy. Science.

[3] Himmel ME, Ding SY, Johnson DK, Adney WS, Nimlos MR, Brady JW, Foust TD (2007). Biomass recalcitrance: engineering plants and enzymes for biofuels production. Science.

[4] Barakat A, Monlau F, Steyer JP, Carrere H (2012). Effect of lignin-derived and furan compounds found in lignocellulosic hydrolysates on biomethane production. Bioresour Technol.

[5] Hsu T, Wyman C (1996). Chap. 10: Pretreatment of biomass. Handbook on Bioethanol: Production and Utilization.

[6] Yang SJ, Kataeva I, Hamilton-Brehm SD, Engle NL, Tschaplinski TJ, Doeppke C, Davis M, Westpheling J, Adams MW (2009). Efficient degradation of lignocellulosic plant biomass, without pretreatment, by the thermophilic anaerobe “*Anaerocellum thermophilum*” DSM 6725. Appl Environ Microbiol.

[7] Blumer-Schuette SE, Giannone RJ, Zurawski JV, Ozdemir I, Ma Q, Yin Y, Xu Y, Kataeva I, Poole FL, Adams MW, Hamilton-Brehm SD, Elkins JG, Larimer FW, Land ML, Hauser LJ, Cottingham RW, Hettich RL, Kelly RM (2012). *Caldicellulosiruptor* core and pangenomes reveal determinants for noncellulosomal thermophilic deconstruction of plant biomass. J Bacteriol.

[8] Basen M, Rhaesa AM, Kataeva I, Prybol CJ, Scott IM, Poole FL, Adams MW (2014). Degradation of high loads of crystalline cellulose and of unpretreated plant biomass by the thermophilic bacterium *Caldicellulosiruptor bescii*. Bioresour Technol.

[9] Chung D, Cha M, Guss AM, Westpheling J (2014). Direct conversion of plant biomass to ethanol by engineered *Caldicellulosiruptor bescii*. Proc Natl Acad Sci U S A.

[10] Vanfossen AL, Verhaart MR, Kengen SM, Kelly RM (2009). Carbohydrate utilization patterns for the extremely thermophilic bacterium *Caldicellulosiruptor saccharolyticus* reveal broad growth substrate preferences. Appl Environ Microbiol.

[11] Zurawski J, Blumer-Schuette S, Conway J, Kelly R, Philippis DZRD (2014). The extremely thermophilic genus *Caldicellulosiruptor*: physiological and genomic characteristics for complex carbohydrate conversion to molecular hydrogen. Microbial BioEnergy: Hydrogen Production, Advances in Photosynthesis and Respiration. Volume 38.

[12] Blumer-Schuette SE, Lewis DL, Kelly RM (2010). Phylogenetic, microbiological, and glycoside hydrolase diversities within the extremely thermophilic, plant biomass-degrading genus *Caldicellulosiruptor*. Appl Environ Microbiol.

[13] Cantarel BL, Coutinho PM, Rancurel C, Bernard T, Lombard V, Henrissat B (2009). The Carbohydrate-Active EnZymes database (CAZy): an expert resource for Glycogenomics. Nucleic Acids Res.

[14] Dam P, Kataeva I, Yang SJ, Zhou F, Yin Y, Chou W, Poole FL, Westpheling J, Hettich R, Giannone R, Lewis DL, Kelly R, Gilbert HJ, Henrissat B, Xu Y, Adams MW (2011). Insights into plant biomass conversion from the genome of the anaerobic thermophilic bacterium *Caldicellulosiruptor bescii* DSM 6725. Nucleic Acids Res.

[15] Bayer EA, Shoham Y, Lamed R, Rosenberg E (2013). Lignocellulose-decomposing bacteria and their enzyme systems. The Prokaryotes - Prokaryotic Physiology and Biochemistry.

[16] Zverlov V, Mahr S, Riedel K, Bronnenmeier K (1998). Properties and gene structure of a bifunctional cellulolytic enzyme (CelA) from the extreme thermophile ‘*Anaerocellum thermophilum*’ with separate glycosyl hydrolase family 9 and 48 catalytic domains. Microbiology.

[17] Chung D, Farkas J, Huddleston JR, Olivar E, Westpheling J (2012). Methylation by a unique alpha-class N4-cytosine methyltransferase is required for DNA transformation of *Caldicellulosiruptor bescii* DSM6725. PLoS One.

[18] Chung D, Farkas J, Westpheling J (2013). Detection of a novel active transposable element in *Caldicellulosiruptor hydrothermalis* and a new search for elements in this genus. J Indust Microbio Biotech.

[19] Clausen A, Mikkelsen MJ, Schroder I, Ahring BK (2004). Cloning, sequencing, and sequence analysis of two novel plasmids from the thermophilic anaerobic bacterium *Anaerocellum thermophilum*. Plasmid.

[20] Chung D, Farkas J, Westpheling J (2013). Overcoming restriction as a barrier to DNA transformation in *Caldicellulosiruptor* species results in efficient marker replacement. Biotech for Biofuels.

[21] Chung DH, Huddleston JR, Farkas J, Westpheling J (2011). Identification and characterization of CbeI, a novel thermostable restriction enzyme from *Caldicellulosiruptor bescii* DSM 6725 and a member of a new subfamily of HaeIII-like enzymes. J Indust Microbio Biotech.

[22] Boeke J, LaCroute F, Fink G (1984). A positive selection for mutants lacking orotidine-5'-phosphate decarboxylase activity in yeast: 5-fluoro-orotic acid resistance. Molec General Genetics.

[23] Miroshnichenko ML, Kublanov IV, Kostrikina NA, Tourova TP, Kolganova TV, Birkeland NK, Bonch-Osmolovskaya EA (2008). *Caldicellulosiruptor kronotskyensis* sp. nov. and *Caldicellulosiruptor hydrothermalis* sp. nov., two extremely thermophilic, cellulolytic, anaerobic bacteria from Kamchatka thermal springs. Int J Syst Evol Microbiol.

[24] Chung D, Cha M, Farkas J, Westpheling J (2013). Construction of a stable replicating shuttle vector for *Caldicellulosiruptor* species: use for extending genetic methodologies to other members of this genus. PLoS One.

[25] O'Sullivan D, Klaenhammer T (1993). Rapid mini-prep isolation of high-quality plasmid DNA from *Lacotococcus* and *Lactobacillus* spp. Appl Environ Microbiol.

[26] Blumer-Schuette SE, Ozdemir I, Mistry D, Lucas S, Lapidus A, Cheng JF, Goodwin LA, Pitluck S, Land ML, Hauser LJ, Woyke T, Mikhailova N, Pati A, Kyrpides NC, Ivanova N, Detter JC, Walston-Davenport K, Han S, Adams MW, Kelly RM (2011). Complete genome sequences for the anaerobic, extremely thermophilic plant biomass-degrading bacteria *Caldicellulosiruptor hydrothermalis, Caldicellulosiruptor kristjanssonii, Caldicellulosiruptor kronotskyensis*, *Caldicellulosiruptor owensensis*, and *Caldicellulosiruptor lactoaceticus*. J Bacteriol.

[27] Farkas J, Chung D, Cha M, Copeland J, Grayeski P, Westpheling J (2013). Improved growth media and culture techniques for genetic analysis and assessment of biomass utilization by *Caldicellulosiruptor bescii*. J Indust Microbio Biotech.

[28] Donahue JP, Israel DA, Peek RM, Blaser MJ, Miller GG (2000). Overcoming the restriction barrier to plasmid transformation of *Helicobacter pylori*. Mol Microbiol.

[29] Johnston C, Martin B, Fichant G, Polard P, Claverys J-PP (2014). Bacterial transformation: distribution, shared mechanisms and divergent control. Nat Rev Microbiol.

[30] Shaw AJ, Hogsett DA, Lynd LR (2010). Natural competence in *Thermoanaerobacter* and *Thermoanaerobacterium* species. Appl Environ Microbiol.

[31] Lee C, Ow D, Oh S (2006). Quantitative real-time polymerase chain reaction for determination of plasmid copy number in bacteria. J Microbiol Methods.

