# Supplementary material for: Heterologous complementation of a pyrF deletion in Caldicellulosiruptor hydrothermalis generates a new host for the analysis of biomass deconstruction
Source: Biotechnol Biofuels. 2014 Sep 16;7:132. doi: 10.1186/s13068-014-0132-8 (PMC4172971; doi:10.1186/s13068-014-0132-8)
Supplement: Additional file 1: Figure S1. — Repair of the pyrF gene in pDCW89 transformants. Figure S2. Evidence for transformation and stable replication of the Caldicellulosiruptor/E. coli shuttle vector pJGW07 in C. hydrothermalis. Figure S3. Restriction digest analysis of pJGW07. Figure S4. Maintenance of the mutated pyrF gene in pJGW07 transformants. Table S1. Quantitative PCR data. Table S2. Primers used in this study. [file 13068_2014_132_MOESM1_ESM.docx]

**Supplemental Figure S1**

M 1 2 3 4

**
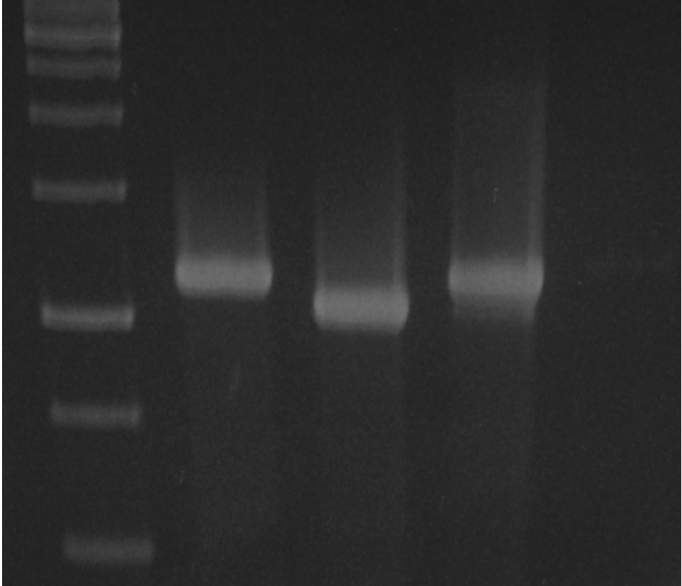
**

Kb

2.5 –

2.0 –

1.5 –

1.0 –

0.75 –

0.5 –

**Figure S1. Repair of the *pyrF* gene in pDCW89 transformants.** *C. hydrothermalis* JWCH006 (*ΔpyrF*) was transformed with M.CbeI-methylated pDCW89, and individual transformant colonies were picked. DNA was isolated from the strain, and PCR using *pyrF*-flanking primers DC163 and DC164 was performed. M: molecular weight standards (NEB); 1: *C. hydrothermalis* wild-type genomic DNA; 2: *C. hydrothermalis* JWCH006 genomic DNA; 3: genomic DNA from *C. hydrothermalis* JWCH006 transformed with pDCW89; 4: negative control.

**Supplemental Figure S2**

**
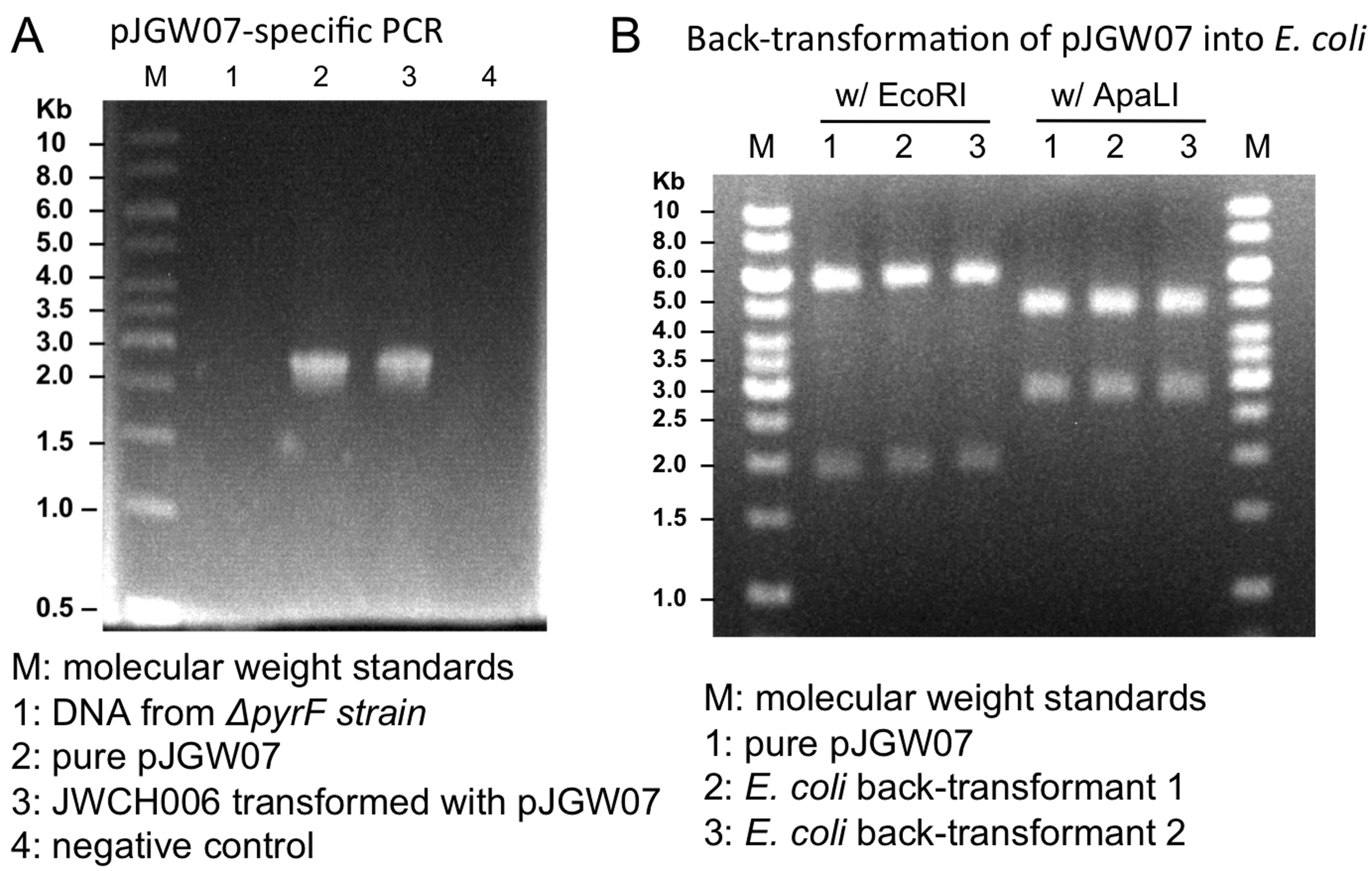
**

**Figure S2. Evidence for transformation and stable replication of the *Caldicellulosiruptor/E. coli* shuttle vector pJGW07 in *C. hydrothermalis.***

**A)** pJGW07 is present in transformants**.** DNA was isolated from the indicated *C. hydrothermalis* strains. PCR was performed using two pJGW07-specific primers JG021 and JF199 (see Fig. 1C) with an expected amplicon of 2.6 kb.

**B)** pJGW07 is structurally stable in *C. hydrothermalis****.*** Back-transformation of pJGW07 isolated from *C. hydrothermalis* into *E. coli* DH5α. For *EcoRI* digests, expected bands: 5.8 kb and 1.9 kb*.* For *ApaLI* digests, expected bands: 5 kb and 2.7 kb (See Fig. 2A). 12 *E. coli* colonies were analyzed, restriction digests of plasmid isolated from two colonies is shown.

**
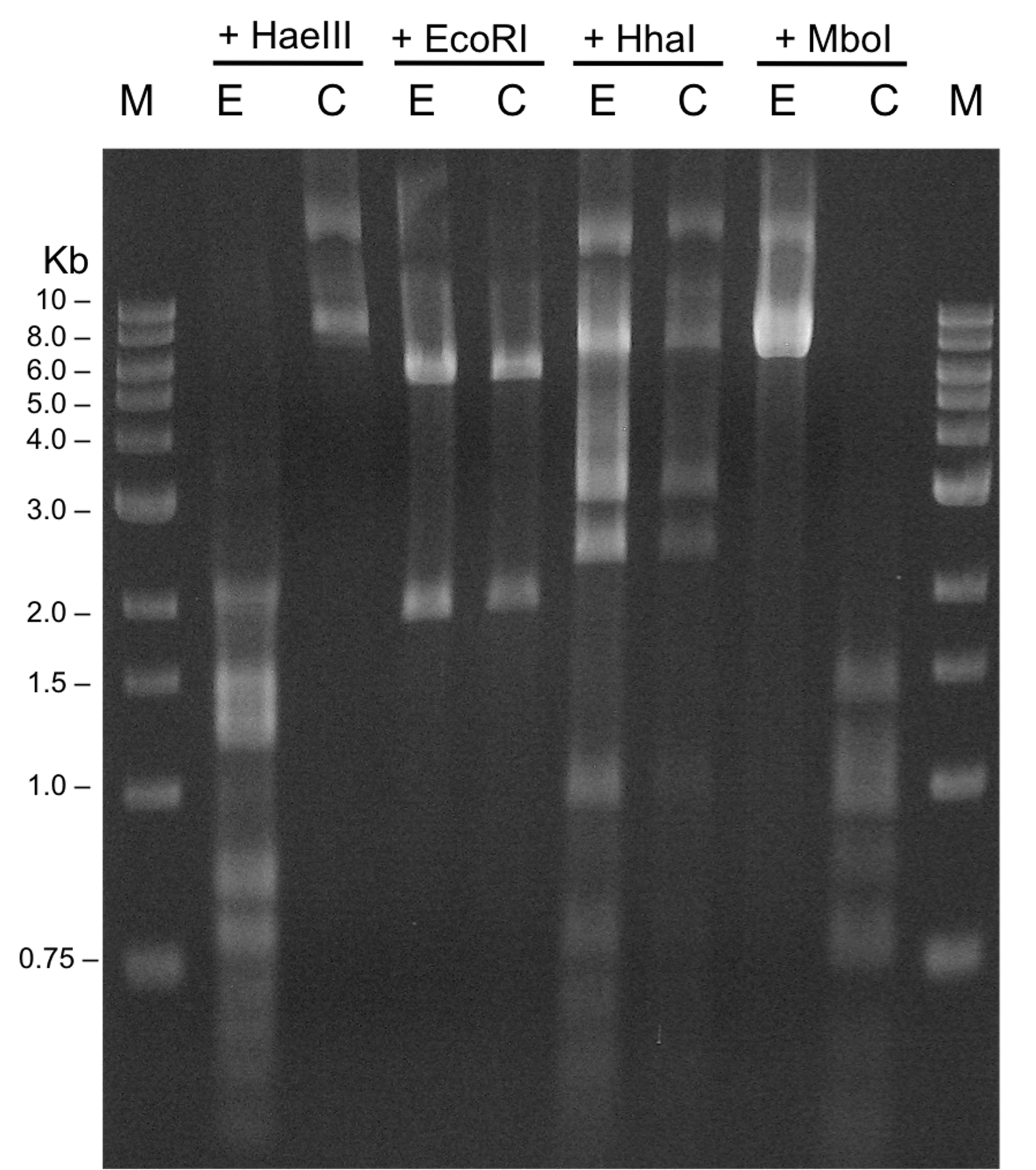
Supplemental Figure S3**

**Figure S3. Restriction digest analysis of pJGW07.** Plasmid pJGW07 purified from either *E. coli* (lanes labeled E) or *C. hydrothermalis* (lanes labeled C) was exposed to enzymes HaeIII, EcoRI, HhaI, or MboI in individual reactions. Electrophoresis profiles of each digest reaction are shown. M, molecular weight standards (NEB).

**Supplemental Figure S4.**

**
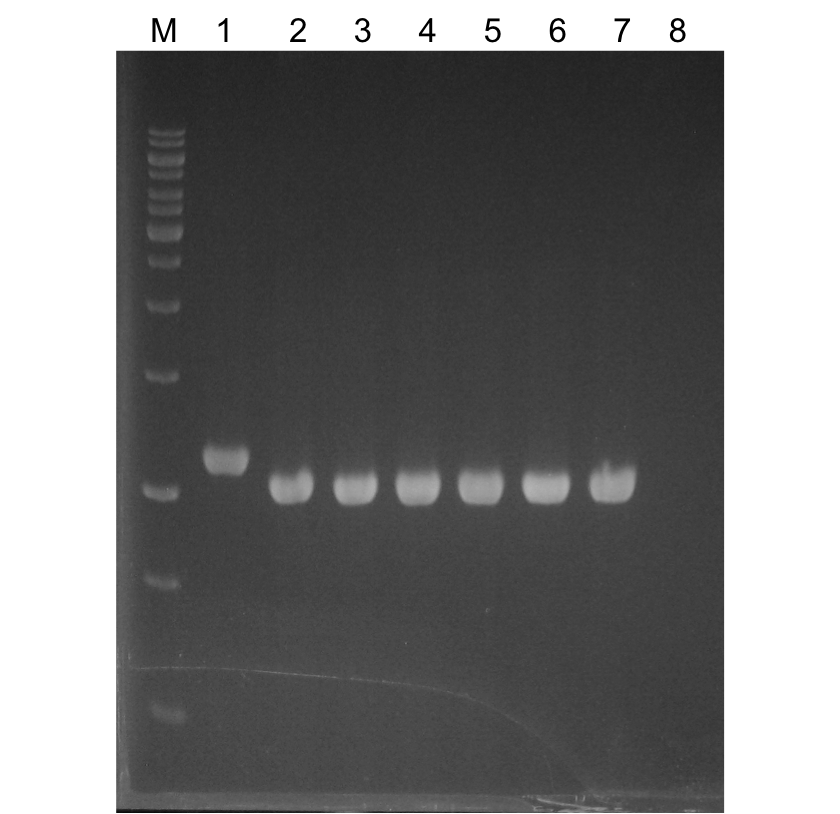
**

Kb

2.5 –

2.0 –

1.5 –

1.0 –

0.75 –

0.5 –

**Figure S4. Maintenance of the mutated *pyrF* gene in pJGW07 transformants.** *C. hydrothermalis* JWCH006 (*ΔpyrF*) was transformed with M.CbeI-methylated pJGW07, and individual transformant colonies were picked. DNA was isolated from the strain, and PCR using *pyrF*-flanking primers DC163 and DC164 was performed. M: molecular weight standards; 1: *C. hydrothermalis* wild-type genomic DNA; 2: *C. hydrothermalis* JWCH006 genomic DNA. 3 through 7: genomic DNA from five individual colonies of *C. hydrothermalis* JWCH006 transformed with pJGW07; 8: negative control.

**Supplemental Table 1: Quantitative PCR data**

| **Value** | **Day 1** | **Day 2** | **Day 3** | **Day 4** | **Day 5** |
| --- | --- | --- | --- | --- | --- |
| Plasmid (Ct_P_) | 21.02 | 21.54 | 20.04 | 19.38 | 18.22 |
| Chromosome (Ct_C_) | 26.74 | 26.38 | 26.08 | 26.24 | 23.99 |
| Ct_c_ – Ct_P_ | 5.72 | 4.84 | 6.04 | 6.87 | 5.78 |
| Copy number (PCN) | 52.77 | 28.54 | 65.72 | 116.84 | 54.76 |

These data represent the amplification cycles required to cross a threshold based on SYBR green fluorescence. qPCR was performed on total DNA isolated from *C. hydrothermalis* over the course of five passages through selective media. Passages through non-selective media resulted in Ct values above 30, comparable with the negative control results, so these values were not interpreted as accurate [[31](#_ENREF_31)]. PCN was calculated using the formula PCN = 2 ^ | Ct_chromosome_— Ct_plasmid_ |. Ct_P_ and Ct_C_ are the average of three replicate qPCR reactions. An amplication efficiency curve over four logs of DNA concentration revealed an efficiency of 93.5%, which is within the acceptable range of 90-110%.

**Supplemental Table 2: Primers used in this study**

| Primer | Sequence |
| --- | --- |
| DC081 | 5′ — AGAGAGGTACCACCAGCCTAACTTCGATCATGGGA — 3′ |
| DC163 | 5′ — TCCTGAACCAATAACCAAAACCT — 3′ |
| DC164 | 5′ — agtgggaagtgaaagaggaaaac — 3′ |
| DC262 | 5′ — TGTGTGGTGCACTCTGACGCTCAGTGGAACGAA — 3′ |
| DC484 | 5′ — AGACTCCGATCGATTCCCATGAGCCCACGAACAGT — 3′ |
| DC485 | 5′ — ATGTGCGATTCCTTTTGCGGTTTGGTCCAT — 3′ |
| DC486 | 5′ — ACCAAACCGCAAAAGGAATCGCACATCGAAAGTTGGGAGT — 3′ |
| DC487 | 5′ — ACAACAGTGCACACTCCATGTAAAGCGATTTTCA — 3′ |
| JF199 | 5′ — TCGCTAACGGATTCACCACT — 3′ |
| JG021 | 5′ — AGAATATCTAGAATGTTTATTGATACATTAATTGAAAAGATTAG AGAAAAGG — 3′ |
| JG022 | 5′ — TGTAGTCCATGGTTACTTCCTGTCTCGCAACGC — 3′ |
| JG023 | 5′ — TCTACTCCATGGTCATCTGTGATATGGACAGTTTTCC — 3′ |
| JG024 | 5′ — AGATCATCTAGAGACCATCCTTTCTATGTAGAAA — 3′ |
| JG025 | 5′ — CTGCCAAGTTAGAAAACAAGGAC — 3′ |
| JG026 | 5′ — AGAACAAGGAATACCAAGCCA — 3′ |
| JG027 | 5′ — ACCTTGCTGTGATAGAAAACCT — 3′ |
| JG028 | 5′ — CATACAATCGGGATTCAGCAGT — 3′ |
| Q1 | 5′ — TGGGAAAGCCGTCCATAATC — 3′ |
| Q2 | 5′ — TCTCCCGCTCTTCTCTCTTT — 3′ |
| Q3 | 5′ — GTGCGTCTACAGGACCTTATTT — 3′ |
| Q4 | 5′ — GGCAAGATTCTACAGGCAAGA — 3′ |
| QH11 | 5′ — CACATCAGCAACAGCAAGTAAG — 3′ |
| QH12 | 5′ — CCTCACAAGCAACTACTCTACC — 3′ |
| QH13 | 5′ — GCTCGGTCGCTCTGAATATAAC — 3′ |
| QH14 | 5′ — GAGTTGGAAAGCTCAGGTCATC — 3′ |
